# Investigation on the Tensile Fracture Properties of Fully-Graded Concrete Considering Aggregate Morphological Characteristics via Peridynamics

**DOI:** 10.3390/ma18163750

**Published:** 2025-08-11

**Authors:** Jie Chen, Houmin Li, Weichao Deng, Keyang Wu, Tianhao Yao, Zhengpeng Zhou, Yunlong Wu

**Affiliations:** 1 School of Civil Engineering, Architecture and the Environment, Hubei University of Technology, Wuhan 430068, China; 2 Innovation Demonstration Base of Ecological Environment Geotechnical and Ecological Restoration of Rivers and Lakes, Hubei University of Technology, Wuhan 430068, China; 3 China Construction Fourth Engineering Division the First Corp., Ltd., Guangzhou 510665, China; 4 Wuhan Construction Engineering Co., Ltd., Wuhan 430014, China; wukeyang@wceg.com.cn

**Keywords:** fully graded concrete, peridynamics, axial ratio, angularity coefficient, uniaxial tensile

## Abstract

Fully graded concrete exhibits significantly enhanced mechanical properties due to optimized aggregate gradation. However, the effects of coarse aggregate morphological characteristics at the microscale—such as axial ratio (Ar) and angularity coefficient (Ac)—on fracture behavior remain insufficiently understood and require further investigation. This study investigates the fracture behavior of fully graded concrete using a peridynamic approach. First, a multilinear constitutive model of concrete considering nonlocal effects and damage evolution is constructed. Second, aggregate modeling via the Tensile-Concave Synthesis Method in peridynamics, and different property bonds and random bond parameters are introduced to characterize the heterogeneity and initial defects of concrete. Finally, the effects of loading rate, aggregate randomness, and the parameters Ar and Ac on the uniaxial tensile properties of concrete are systematically studied. The results demonstrate that peridynamics accurately captures the entire process of crack initiation and propagation in fully graded concrete. Uniaxial tensile behavior exhibits strong rate dependence. Different random aggregate models result in variations in the peak tensile strength of concrete. With the increase in Ar, the peak tensile stress gradually decreased by 4.15%, whereas the elastic modulus increased by 14.31%. As Ac increased, the peak stress exhibited an overall trend of first increasing by 5.14%, followed by a decrease of 3.8%. Therefore, in numerical simulations, the influences of loading rate and aggregate randomness should not be overlooked. Moreover, to enhance the strength of fully graded concrete, the proportion of aggregates with large Ar and Ac values should be minimized.

## 1. Introduction

Concrete used in dam engineering typically employs fully graded or three-graded aggregate systems [1]. This composite combines coarse aggregates with a cementitious matrix, water, and admixtures in optimized proportions. Upon curing under controlled temperature and humidity, it forms a hardened structure that serves as a key material in modern hydraulic structures. Engineering practice has shown that load-bearing concrete components exhibit brittle fracture behavior under tensile stress. Thus, tensile strength parameters are critical for structural design [2]. Microstructural analysis reveals that concrete is a typical multi-phase composite material consisting of coarse aggregates, a cement mortar matrix, pores, and an interfacial transition zone (ITZ) [3]. The complex material morphology induces substantial geometric heterogeneity in mechanical properties. Especially in mass hydraulic concrete, the aggregate shape tends to be more complex, and the maximum aggregate size typically exceeds 80 mm, further exacerbating the heterogeneity of the internal material structure. This heterogeneity leads to stochastic nonlinearity in crack initiation and propagation, complicating the systematic analysis of fracture mechanisms [4].

Experimental testing remains an essential approach for investigating the fracture behavior of concrete. However, physical model testing of hydraulic concrete prototypes—incorporating fully graded aggregates and large-scale specimens—requires extensive facilities, significant material resources, and highly specialized equipment, which poses significant challenges for routine implementation.

With advances in computational technology, numerical simulations can now model the complex mechanical behavior and failure mechanisms of concrete at the microscale under coupled physical fields. By accurately characterizing bond parameters—including mortar matrix distribution, geometric features of coarse aggregates, ITZ properties, and initial microdefects—and integrating high-performance numerical simulations with experimental validation, microscale models can effectively capture internal damage accumulation and crack network evolution during fracture. This multi-physics coupling analysis provides a theoretical foundation for understanding the mechanisms underlying the variability in the fracture performance of hydraulic concrete [5,6,7]. To investigate the effects of concrete mesostructure—such as pores and microcracks—on failure behavior, researchers have widely employed numerical methods based on classical continuum mechanics, including the Finite Element Method (FEM) [8], Finite Difference Method (FDM) [9], Boundary Element Method (BEM) [10], and Finite Volume Method (FVM) [11]. These methods have become mainstream tools in the field due to their implementation convenience, computational efficiency, and applicability in engineering practice. However, the presence of stress singularities near crack tips often results in poor convergence in these regions, limiting the accuracy of damage accumulation and crack propagation predictions. Consequently, specialized numerical techniques are required to address discontinuities. To more effectively simulate crack growth, various advanced methods have been proposed. For instance, the Extended Finite Element Method (XFEM) [12] allows cracks to propagate independently of the mesh within elements, Cohesive Zone Model (CZM) [7] simulates fracture processes by introducing interface elements along potential crack paths, and Phase Field Method (PFM) [13] describes the evolution of crack topology in a diffused manner through an auxiliary phase field variable. Despite their respective advantages, these methods still face significant trade-offs between computational accuracy and efficiency when applied to complex concrete fracture problems.

Peridynamics (PD), proposed by Silling in 2000, provides a fundamentally different nonlocal theoretical framework [14]. Its core concept lies in replacing the spatial derivatives used in classical continuum mechanics with integral-form equations of motion. This fundamental shift enables PD to naturally avoid the mathematical and numerical difficulties encountered by traditional methods in dealing with stress singularities at crack tips, spontaneous crack initiation, and arbitrary crack path propagation. Compared with local continuum-based methods (such as FEM, FDM, BEM, and FVM) and enhanced techniques that require predefined crack paths or additional degrees of freedom/phase-field variables (e.g., XFEM, CZM, PFM), PD offers significant theoretical advantages in modeling material discontinuities, especially for complex fracture behaviors. Recent advances highlight the exceptional capability of peridynamics in modeling the fracture mechanisms of hydraulic concrete structures, particularly in capturing spontaneous crack propagation and multiscale damage evolution. For instance, Li et al. [15] developed a peridynamics model to simulate microcrack propagation in concrete. They systematically investigated fracture mechanisms under tensile, compressive, and shear loading conditions. Zhang et al. [16] proposed a coupled water–thermal–mechanical (WTM) approach to analyze freeze–thaw-induced cracking in concrete, effectively capturing both the cracking process and the underlying failure mechanisms. Gu et al. [17] developed a peridynamic model to investigate quasi-static deformation and projectile impact-induced damage in concrete gravity dams. These studies demonstrate that peridynamics, as an advanced theoretical framework, has been widely applied to investigate the mechanical behavior of concrete, showing distinct advantages in modeling and simulating complex failure processes. Its scientific validity and reliability have been widely acknowledged by experts in the field.

However, due to the complexity of modeling concrete at the microscale, most existing studies have focused on fine-scale structures, leading to the development of various fine-scale models. Current research primarily adopts two computational approaches: the Intermediate Homogenization Model (IH-PD) [18,19,20] and the Mesoscale Modeling framework [21,22,23]. Through systematic evaluation of microstructural mechanical characterization, Wu et al. [24] demonstrated that fine-scale computational approaches offer superior predictive capabilities compared to intermediate homogenization methods. As a result, these methods have seen broader adoption in concrete modeling. However, researchers frequently employ simplified circular aggregate geometries when implementing such frameworks [21,22,23,24,25]. While circular aggregate idealization remains prevalent in fine-scale modeling, excessive geometric simplification may compromise a model’s ability to capture multiscale mechanical responses of cementitious composites. Previous studies have demonstrated that aggregate morphology is a critical factor influencing the mechanical properties of fully graded concrete [26,27]. However, in meso-scale modeling of aggregate geometry in concrete, random polygonal shapes are commonly used, which do not accurately capture the actual morphological characteristics of aggregates [6,7,13]. Hence, an accurate investigation of fully graded concrete’s mechanical behavior requires advanced modeling frameworks capable of quantifying aggregate morphological parameters.

Therefore, to overcome the limitations of existing numerical simulation methods and fill the research gap regarding the influence of aggregate morphology on the uniaxial tensile damage mechanism of fully graded concrete at the meso-scale, this study develops a meso-scale model of fully graded concrete that incorporates both aggregate morphology parameters and random heterogeneity. A corresponding uniaxial tensile PD computational framework is established to evaluate the effects of aggregate morphology parameters on the mechanical properties of fully graded concrete under uniaxial tensile loading, thereby providing theoretical support for optimizing aggregate morphology in fully graded concrete. Section 2 introduces the basic PD theory and the multilinear principal structure establishment method. Section 3 presents the establishment method of a calculation model for fully graded concrete with morphological characteristics. Section 4 discusses and analyzes the effects of loading rate, aggregate randomness, aggregate axis length ratio, and angularity coefficient on the uniaxial tensile strength and fracture behavior of fully graded concrete.

## 2. Theoretical Background of Peridynamics

### 2.1. Basic Theory

**Figure 1:**
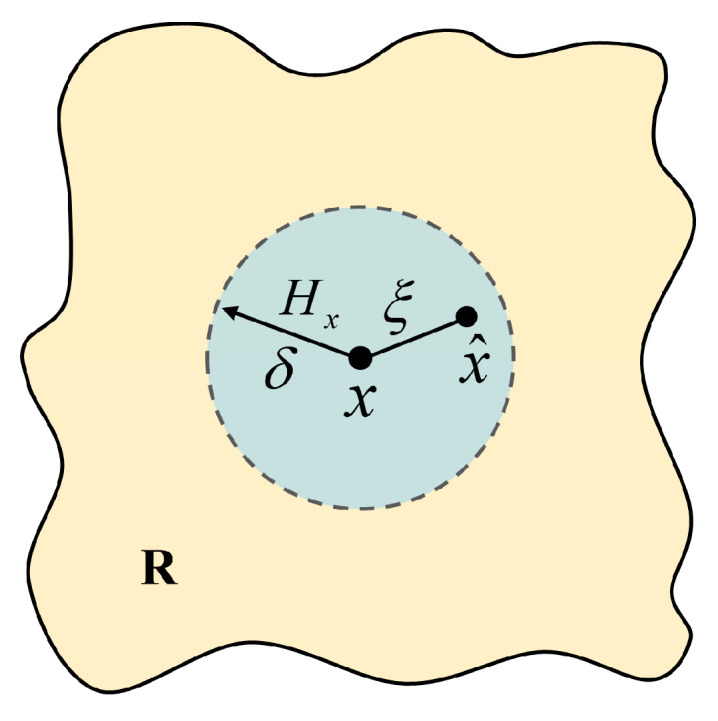
Schematic diagram of substance point x in family $H_{x}$.

Peridynamics (PD), a nonlocal continuum formulation pioneered by Silling [14], employs integral operators to describe interparticle interactions, fundamentally differing from classical continuum mechanics, which relies on partial differential equations. It assumes that matter consists of an infinite number of discrete material points, each possessing a finite mass and volume. In bond-based peridynamics (BBPD), the interaction forces between particle points are collinear, opposite in direction, and equal in magnitude. In non-local methods, particles interact with all other particles $\hat{x}$, within a horizon radius δ or peridynamics range $H_{x}$ through so-called “bonds.” Intact materials that have not undergone damage or destruction interact with their neighboring material points through “bonds.” As shown in Figure 1, material point x and material point $\hat{x}\;$ are connected by bond ξ.

The basic equation of PD theory is as follows:

(1) $$\rho \left(x\right)\ddot{\upsilon_{x}}\left(t\right)=\int_{H\left(x\right)}f\left(u\left(\hat{x},t\right)-u\left(x,t\right),\hat{x}-x\right)\;dV_{\hat{x}}+b\left(x,t\right)$$

where $\rho \left(x\right)$ is the density of particle point x, ${\ddot{u}}_{x}$ represents the acceleration of the material particles at time t, u represents the displacement vector of the particle point, $dV_{\hat{x}}$ is the volume element of particle point $\hat{x}$, $b\left(x,t\right)$ is the physical force function of the particle point, and f is the force vector between particle points.

**Figure 2:**
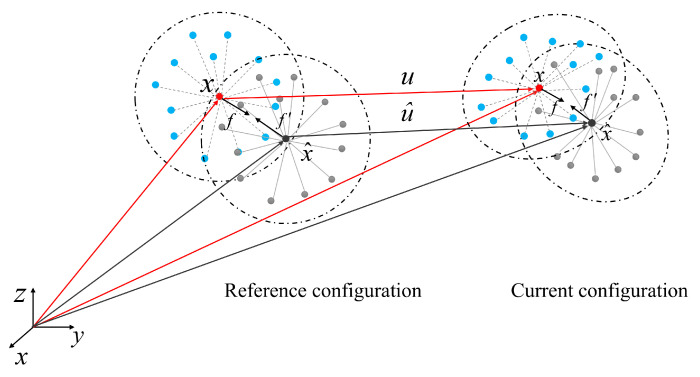
Schematic diagram of the interaction between two material points x and $\hat{x}$.

Figure 2 illustrates the kinematic relationship between a pair of material points in reference and current configurations. In peridynamic theory, the interaction force within the horizon radius for homogeneous materials is described by a displacement-dependent function, in which the bond force magnitude is determined by the spatial coordinates in the reference configuration and the relative displacement vector. Let the relative position vector between material points be defined as *ξ*$=x-\hat{x}$, and the relative displacement vector as $\eta =u-\hat{u}$. For the circular near-field region, considering the Prototype Micro-elastic Brittle (PMB) material [14], the bond force can be expressed as shown in Equations (2)–(4).

(2) $$f\left(\eta ,\xi \right)=\left\{\begin{array}{c} \frac{\xi +\eta }{\left|\xi +\eta \right|}cs\mu \;\;\;\;\;\;\;\left(\xi \leq \delta \right)\;\\ 0\;\;\;\;\;\;\;\;\;\;\;\;\;\;\;\;\;\;\;\;\;\;\;\;\;\left(\xi >\delta \right) \end{array}\;\right.$$

(3) $$c=\frac{12k}{\pi \delta^{3}}$$

(4) $$s=\frac{\left|\xi +\eta \right|-\left|\xi \right|}{\left|\xi +\eta \right|}$$

where c is the peridynamics microelastic constant characterizing the bond stiffness, s is the bond tensile strength, μ  is a historical dependence scalar function characterizing bond damage, and k is the bulk modulus of the material in a two-dimensional plane.

The energy release rate of PMB materials can be related to the ultimate bond elongation using Equations (5) and (6).

(5) $$G_{1}=\int_{0}^{\delta }\int_{H\left(z\right)}\omega_{1}\;dV\;dz=\frac{cs_{1}^{\;2}S^{4}}{4}$$

(6) $$s_{1}=\sqrt{\frac{\pi G_{1}}{3k^{\prime }\delta }}$$

where $\omega_{1}$ is the energy density of a single bond (i.e., the ratio of absorbed energy to the volume of material points), $H_{\left(z\right)}$ is the near-field range on the other side of the crack, and z is the distance from the material point to the crack surface.

### 2.2. Multi-Segment Linear Constitutive Theory

**Figure 3:**
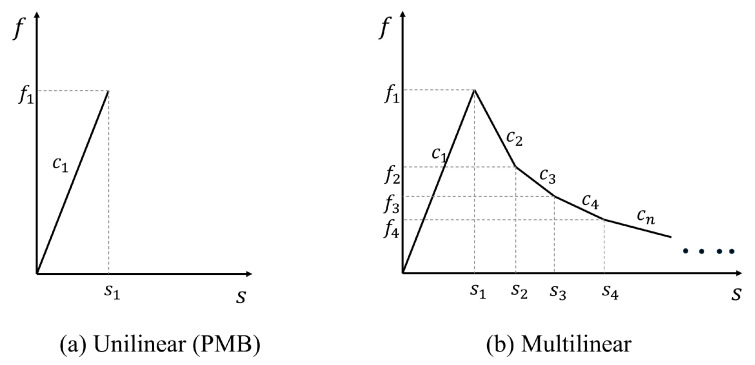
Relationship between force density and elongation for single-linear (PMB) and multi-segment linear bonds.

The bond-based peridynamics (BBPD) framework employs a linear damage criterion specifically developed for PMB materials. As shown in Figure 3a, this constitutive relationship defines bond rupture when the normalized stretch ratio s reaches a critical threshold $s_{1}$. Although this constitutive formulation offers computational efficiency, its oversimplified nature limits its ability to capture the progressive damage evolution and multi-scale failure characteristics inherent to quasi-brittle materials such as concrete. Concrete exhibits a distinct stress-softening stage during tensile failure. The PMB model exhibits unilinear behavior but is incapable of simulating stress-softening. This study introduces a modified PMB fracture model designed to more accurately simulate the uniaxial tensile failure behavior of concrete. The proposed model employs a multi-segment linear approximation to represent the stress-softening behavior of concrete under tensile fracture conditions. The force-elongation relationships of multi-segment linear elements are illustrated in Figure 3b.

**Figure 4:**
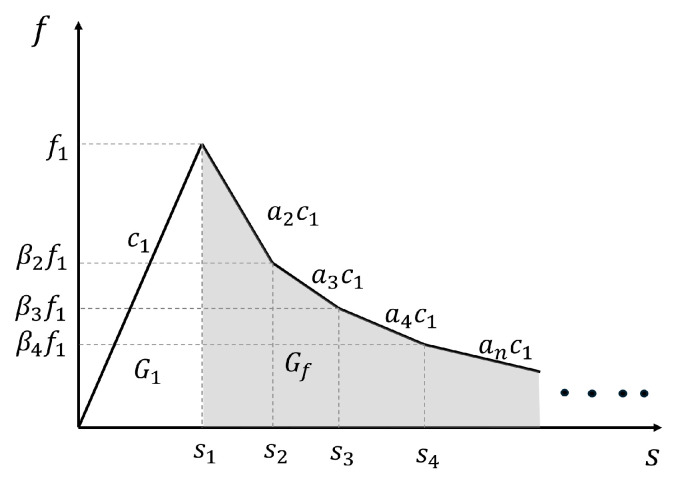
Method for controlling the force density-elongation ratio of multi-segment linear bonds.

In defining the segments of the multi-linear model, it is understood that the cohesive zone model (CZM) uses a bilinear softening constitutive curve [28], with the softening region typically assumed to correspond to the fracture energy of concrete. Later, based on the findings of Wu et al. [29], the full stress–strain curve of concrete is composed of multiple stages, each associated with varying tensile behaviors and energy release mechanisms. Incorporating the ASTM C1018 toughness index method [30], the terminal strain is defined as a multiple of the strain at the initial cracking point, allowing each segment’s strain to be represented as a multiple of this reference value. Drawing from the above research, the concrete softening model can be partitioned into a finite number of segments according to the magnitude of energy release, using the elastic segment to regulate the parameters of the following segments. Thus, in the multi-linear model established in this study, the spring stiffness in the elastic stage is determined using the same method as in the bond-based peridynamics (BBPD) framework. For the following stages, the slope in each segment of the multi-linear model is governed by the ratio between the current descending slope and the spring stiffness of the elastic segment. Simultaneously, the width of each interval on the elongation axis is determined by the ratio between the reduction in force density for each linear segment and the peak force density of the elastic segment, as shown in Figure 4.

In the linear segment control method, the work performed by interparticle interaction forces within each linear segment is governed by three parameters: the spring constant of the elastic segment, the maximum force density, and the initial interparticle spacing. The energy density and corresponding elongation rate at each stage are calculated using Equations (7) and (8), respectively.

(7) $$\omega_{i-i+1}=\frac{f_{i}+f_{i+1}}{2}\left(s_{i+1}-s_{i}\right)\xi =\frac{\left(\beta_{i}+\beta_{i+1}\right)\left(\beta_{i}-\beta_{i+1}\right)}{\alpha_{i}}\frac{c_{1}s_{1}^{2}}{2}\xi$$

(8) $$s_{i}=\frac{\left(\beta_{i}-\beta_{i+1}\right)}{\alpha_{i}}\sqrt{\frac{\pi G_{1}}{3k^{\prime }\delta }}+s_{i-1}$$

where $\omega_{i-i+1}$ is the energy density absorbed by the bond in interval i−i+1, $a_{i}$ is the ratio of the absolute value of the slope of the force density function decrease in the *i*-th interval $c_{i}$ to the slope of the elastic interval force density function increase $c_{1}$, where $a_{1}=1$; $\beta_{i}$ is the ratio of the force density function $f_{i}$ at the *i*-th interval boundary point to the force density function $f_{1}$ at the elastic segment boundary point, where $\beta_{1}=1$. a is the elongation rate of the *i*-th interval bond, where $s_{1}$ is calculated using Equation (6).

It can be observed that under constant interparticle spacing, the ratio of the energy required for bond rupture between the multi-segment linear model and the single-segment linear model—both with identical spring constants and ultimate elongation values—remains invariant with respect to variations in the elastic segment spring constant or ultimate elongation, as illustrated in Equation (9). Accordingly, the overall energy density can be calculated as shown in Equation (10).

(9) $$\gamma_{i}=\frac{\left(\beta_{i}+\beta_{i+1}\right)\left(\beta_{i}-\beta_{i+1}\right)}{\alpha_{i}}$$

(10) $$\omega_{w}=\sum_{i=0}^{n}\gamma_{i}\omega_{0-1}=\gamma \omega_{0-1}$$

where $\omega_{w}$ is the energy density required to break the bond in the multi-segment linear state, $\omega_{0-1}$ is the energy density absorbed in the first interval, $\gamma_{i}$ the ratio of the energy density absorbed by the bond in the *i*-th interval and $\omega_{0-1}$, and γ is the ratio of $\omega_{w}$ and $\omega_{0-1}$. In the two-dimensional case, the fracture energy $G_{f}=(\gamma -1)G_{1}$ of the post-peak (descent) stage can be determined by calculating the ratio of the energy density at each stage to that of the elastic stage.

When the multi-linear quasi-brittle damage model, which is based on a single-linear improvement, is implemented in the BBPD framework, the bond weakening is described by a scalar value function μ(s,t) defined in Equation (11), with values ranging from 0 to 1.

(11) $$\mu \left(s,t\right)=\left\{\begin{array}{c} 1\;\;\;\;\;\;\;\;\;\;\;\;\;\;\;\;\;\;\;\;\;s\leq s_{1}\\ \frac{\left|f\left(\eta ,\xi \right)\right|}{c_{1}s_{1}}\;\;\;\;\;\;\;\;s_{1}<s<s_{n}\\ 0\;\;\;\;\;\;\;\;\;\;\;\;\;\;\;\;\;\;\;\;\;\;\;\;\;\;\;s\geq s_{n} \end{array}\right.$$

Further, the damage of peridynamics is defined as the ratio of the number of broken bonds to the total number of bonds in the near-field range of the material point.

(12) $$D\left(x,t\right)=1-\frac{\int_{H\left(x\right)}\mu \left(s,t\right)dV_{\hat{x}}}{\int_{H\left(x\right)}dV_{\hat{x}}}$$

This scalar function quantifies the degree of damage at a point and takes values from 0 to 1, with 0 indicating that no bond-breaking damage has occurred at the point and 1 indicating that all bonds between the point and all other material points in its horizon are broken.

## 3. Constructing a Fully Graded Concrete Model Considering Aggregate Morphology Characteristics

### 3.1. Parameters of Morphological Characteristics of Aggregates

**Figure 5:**
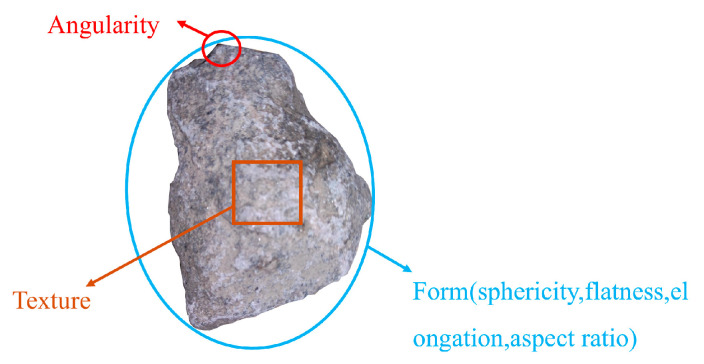
Morphological characteristics of aggregates.

Concrete consists of aggregate particles embedded in a hydrated cement paste matrix, exhibiting inherent heterogeneity. Aggregate morphology—including particle geometry and maximum size distribution—as well as spatial arrangement, plays a critical role in governing the macroscopic mechanical behavior of concrete. Aggregates are typically produced by mechanically crushing and screening natural rock, resulting in particles with irregular polygonal shapes. Aggregate morphology can be simultaneously decomposed into three separate features: Form, Angularity, and Texture [31], each representing different scales of morphological features on the aggregate, as illustrated in Figure 5.

This study establishes a modeling method that incorporates aggregate morphological characteristics. Kuo [32] and Gu [33] used digital image correlation (DIC) technology to measure aggregate shape parameters and angularity indices under two-dimensional conditions. Specifically, the aspect ratio and angularity coefficient can effectively represent how the aggregate’s shape and angularity affect the mechanical behavior of concrete. Therefore, these two parameters can accurately reflect the geometric morphology of aggregates in a two-dimensional plane. Considering the feasibility of subsequent aggregate generation, this study adopts the aspect ratio of the equivalent ellipse as the shape descriptor. and uses the angularity coefficient as a descriptor for the edge angular features of aggregates. Their mathematical definitions are as follows:

(13) $$\mathrm{Ar}=\frac{l}{b}$$

(14) $$\begin{array}{c} Ac={\left(\frac{P_{p}}{P_{e}}\right)}^{2} \end{array}$$

where Ar is the axial ratio of the aggregate, Ac is the angularity coefficient of the aggregate; l and b are the lengths of the long and short axes of the equivalent ellipse of the aggregate geometry, respectively, and $P_{p}$ and $P_{e}$ are the perimeter of the aggregate geometry and the perimeter of the equivalent ellipse of the aggregate, respectively.

### 3.2. Modeling Aggregates with Axial Ratio and Angularity Coefficient

**Figure 6:**
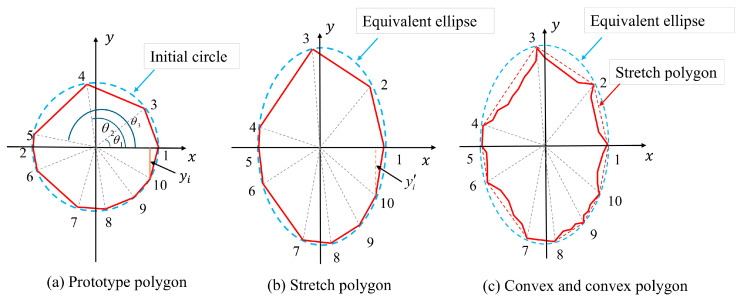
Process of establishing the aggregate model.

This section presents a computational framework for generating aggregates with controlled axial ratio (Ar) and angularity coefficient (Ac). To ensure that the properties of the generated aggregate are closely match the target properties, the aggregate generation method satisfy the following functional requirements: (1) The particle size of the generated aggregate should closely approximate or match the specified value; (2) The morphological parameters of the aggregate—axial ratio and angularity coefficient—within the aggregate group should be distributed close to the specified axial ratio and angularity coefficient values; (3) The geometric shapes of the aggregates should exhibit a certain degree of randomness. To meet these requirements, the aggregate generation process is as follows:

1. Prototype polygon vertex count determination: Aggregate morphological parameters are correlated with variations in the number of polygonal variations in particle geometry [34]. The generation of prototype polygons is driven by specified morphological parameter inputs. As shown in Figure 6a, circular shapes centered at a fixed origin are generated based on the specified morphological parameters. The number of vertices in the prototype polygon varies depending on the interval of Ac, as shown in Equation (15).
  (15) $$\left\{\begin{array}{c} n=6\;\;\;\;\;\;\;\;\;\;\;\;\;\;\;\;\;\;\;\;\;\;\;\;\;\;\;\;\;Ac<1.15\\ n=10\;\;\;\;\;\;\;\;\;\;\;\;\;\;1.15\leq Ac<1.26\\ \begin{array}{c} n=5\;\;\;\;\;\;\;\;\;\;\;\;\;\;\;\;1.26\leq Ac<1.35\\ n=4\;\;\;\;\;\;\;\;\;\;\;\;\;\;\;\;\;\;\;\;\;\;\;\;\;\;\;\;\;Ac\geq 1.35 \end{array} \end{array}\right.$$
2. Determining the angle during aggregate generation: As shown in Figure 6a, when starting to construct a polygon, two vertices of the polygon are fixed at the center angle of 0 and π, and the remaining vertices are randomized using Equation (16).
  (16) $$\theta_{i}=\frac{2\pi }{n}\pm \left(2\psi -1\right)\times \alpha \times \frac{2\pi }{n}$$
  where ψ is a random number uniformly distributed between 0 and 1, and α is any value not greater than 1. The generated $\theta_{i}$ must satisfy Equation (17).
  (17) $$\left\{\begin{array}{c} \Delta \theta_{min}=min\left(\left|\theta_{i}-\theta \right|,\left|\theta_{i}-\theta -2\pi \right|,\left|\theta_{i}-\theta +2\pi \right|\right)\\ \Delta \theta_{min}<\theta_{min} \end{array}\right.$$
  where θ is the angle value that has been determined to have been generated, $\theta_{min}$ is the minimum allowable angle interval, $\left|\theta_{i}-\theta \right|$ is the direct angle difference, $\left|\theta_{i}-\theta -2\pi \right|$ is the 2π difference value of the angle crossing, and $\left|\theta_{i}-\theta +2\pi \right|$ is the difference value of the angle crossing −2π.
3. Determination of aggregate stretching values: Calculate the vertex coordinates $\left(x_{i},y_{i}\right)$ of the prototype polygon using the characteristic angle and the radius of the circumscribed circle. The polygon is then stretched along the y-axis based on the reference axis length ratio. The coordinates of individual polygon vertices are further adjusted using Equation (18), as illustrated in Figure 6b.
  (18) $$y_{i}^{\prime }=S_{c}\cdot y_{i}$$
  Here, $S_{c}$ denotes the vertical magnification factor, which is restricted to the range of [1.0, 3.5]. Based on the prescribed axis length ratio, an appropriate magnification factor within this interval is determined using the bisection algorithm. Next, the equivalent axis length ratio of the aggregate’s fitted ellipse is computed, as illustrated by the blue dotted line in Figure 6b. If the required axis length ratio is not achieved, the stretching process is repeated.
4. Determining the aggregate indentation depth: During the indentation process, the initial indentation depth is uniformly distributed within the range [0.03l,0.1l], where l denotes the side length of the polygon. The rules for identifying concave points on a polygon are defined as follows: let L denote the length of the longest side, and l the length of the current side. When l<0.3L, no concave points are placed on the side; when 0.3L≤l<0.8L, four concave points are placed on the side and divided into five equal parts according to the current side length; when l≥0.8L, eight concave points are placed on the side and divided into nine equal parts according to the current side length. The actual indentation depth of the aggregate polygon is obtained by multiplying the initial indentation depth by the indentation depth magnification factor. Figure 6c illustrates the comparison of the aggregate concavity before and after indentation. As the axis length ratio increases, the distance between the polygonal sides on either side of the aggregate’s long axis decreases. This results in an increased likelihood of edge intersections and excessively small internal angles, which in turn affect the efficiency and robustness of program execution. Therefore, the range of values for the concavity coefficient must be adjusted based on the vertical magnification factor. The minimum and maximum values of the concavity coefficient for each interval are presented in Table 1.

**Figure 7:**
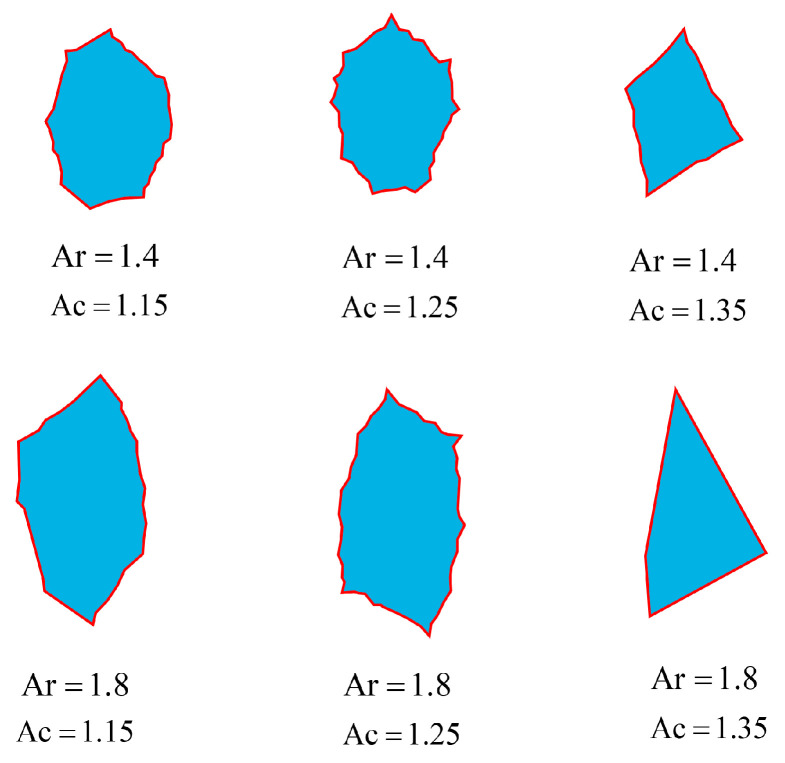
Geometric shapes of aggregates with different morphological characteristics coefficients.

Using the method described above, aggregates with two morphological characteristics—axis length ratio and angularity—can be constructed. This method is based on a prototype polygon, where stretching deformation is applied first, followed by boundary indentation processing; therefore, it is named the Tensile-Concave Synthesis Method. Compared with aggregate models generated by the conventional random polygon method, the random polygon approach does not involve explicit input of aggregate morphology parameters, making it difficult to accurately capture the geometric features and boundary contours of real aggregates in a two-dimensional plane. As shown in Figure 7, aggregate models with different morphological characteristics were generated using the stretching-indenting composite method, with the target morphology parameters set to Ar = 1.4 and Ac = 1.15. A total of 100 aggregate samples were generated, and the mean and standard deviation of Ar and Ac were calculated. The average and standard deviation of Ar were 1.4008 and 0.0087, respectively, while those of Ac were 1.1503 and 0.0060, respectively. The results indicate that this method has good control over morphology parameters and can accurately reproduce the target 2D shape features of aggregates.

### 3.3. Establishment of a Concrete Micro-Scale Model

#### 3.3.1. Concrete Fine-Scale Model Generation and Discretization

In this study, a concrete micromechanical model is developed using the generate-place-assign method. The method comprises three main steps: generating aggregate information with dual morphological characteristics based on the Walraven formula, placing the aggregates within the concrete domain, and assigning corresponding properties based on the location of the material points.

**Figure 8:**
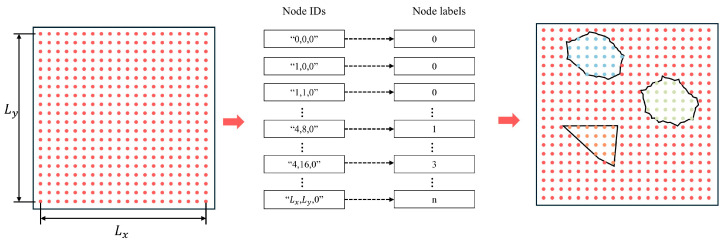
Node marking method.

1. To achieve equivalence with 3D planes, Walraven adapted Fuller’s gradation formula into a two-dimensional aggregate gradation formula, which is more suitable for developing a 2D concrete micro-model, as shown in Equation (19) [8].
  (19) $$P\left(D<D_{i}\right)=P_{k}\left[1.065{\left(\frac{D_{i}}{D_{max}}\right)}^{0.5}-0.053{\left(\frac{D_{i}}{D_{max}}\right)}^{4}-0.012{\left(\frac{D_{i}}{D_{max}}\right)}^{6}-0.0045{\left(\frac{D_{i}}{D_{max}}\right)}^{8}-0.0025{\left(\frac{D_{i}}{D_{max}}\right)}^{10}\right]$$
  where $P\left(D<D_{i}\right)$ represents the area fraction of aggregate particle size D smaller than $D_{i}$, $D_{i}$ is the specified particle size of coarse aggregate, $p_{k}$ is the percentage of coarse aggregate volume in the total concrete volume, and $D_{max}$ is the maximum particle size of aggregate.
  The aggregate particle sizes within a given gradation $\left[D_{i},D_{i+1}\right]$ can be expressed as $D=\left\{D_{1},D_{2},D_{3}\ldots D_{n}\right\}$(where $D_{n}\subset \left[D_{i},D_{i+1}\right]$). These sizes are determined using the initial circular area method. A random particle size D is selected from the aggregate size set to compute the vertex coordinates of the corresponding polygonal aggregate. The polygons in this study are generated using the Tensile-Concave Synthesis Method. The area of each generated aggregate is calculated to verify whether it falls within the specified range. If this condition is not met, the generation process is repeated until the requirement is satisfied.
2. After all aggregates are generated, they are sorted in descending order of size so that larger particles are placed first. When the number of aggregates is large, a comprehensive algorithm is employed to efficiently detect potential intersections. To reduce computational cost, the polygons are first enclosed within axis-aligned bounding boxes (AABB). If two aggregates are determined not to intersect, three possible situations may arise: intersection, separation, and overlap. The surrounding-number method [35] is subsequently used to determine whether a point lies inside the polygon, enabling the classification of the three aforementioned cases.
3. Following aggregate placement within the designated domain, the node-tagging method is employed to assign material point attributes. This method involves calculating both the displacement and tag value of all material points within the domain. For example, as shown in Figure 8, $N_{x}\times N_{y}$ nodes are uniformly distributed over a two-dimensional domain of size $L_{x}\times L_{y}$. Each node in the area is assigned a Node ID based on its coordinate value to prevent errors when assigning material properties. Node IDs are further categorized based on the material phase. All initial tags within an area are set to 0 (mortar area, red), and then different aggregates are marked as “1, 2, 3, 4, &, n”, where n is a positive integer (aggregate area, other colors), indicating that there are n aggregates in the model.

#### 3.3.2. Methods for Establishing Internal Bonds in Concrete

**Figure 9:**
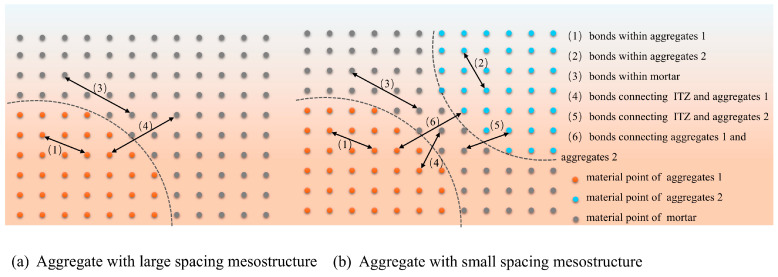
Establishment of corresponding bonds for sparse and dense aggregates.

From a mesoscale perspective, concrete can be regarded as a heterogeneous composite material composed of aggregate and a mortar matrix [36]. By defining interaction bond parameters between material points, distinct material properties are assigned to the coarse aggregate, mortar matrix, and the interfacial transition zone (ITZ). Studies by Guo et al. [6] have demonstrated that fully graded concrete exhibits higher coarse aggregate density than conventional concrete. When establishing concrete mesoscale models, the simplified method developed by Dong et al. [21] can effectively configure bond parameters in conventional models with low aggregate density, as illustrated in Figure 9a. However, under high aggregate density conditions, reduced inter-aggregate spacing (below the near-field threshold) leads to the formation of bond type 6 characteristics between adjacent aggregates, as illustrated in Figure 9b. In mesoscale modeling, bonds between aggregates are typically assigned high-strength parameters, which influence crack propagation paths and directly determine the mechanical response in numerical simulations.

To address this limitation, we propose a mesoscale modeling approach that systematically configures interface bonds between aggregates using differentiated bonding criteria, as illustrated in Figure 9b. This framework defines four distinct bond types for closely spaced aggregates: (1) intra-aggregate, (2) intra-mortar, (3) ITZ, and (4) inter-aggregate bonding. This methodology effectively mitigates artifacts arising from aggregate proximity in PD simulations of crack propagation.

#### 3.3.3. Setting Random Defects Inside Concrete

Inherent microdefects in concrete give rise to spatial heterogeneity in mechanical and physical properties—such as strength and elastic modulus—resulting in non-uniform bond elongation rates and pronounced randomness in material behavior. Various researchers have proposed alternative methods to incorporate mechanical property randomness into material models. Zheng et al. [26] employed the Weibull statistical distribution function to characterize the heterogeneity of concrete internal material properties and introduced a spatial correlation coefficient to consider the correlation and continuity of materials in space. Silling et al. [37] implemented stochastic variations in critical stretching through a Weibull distribution. The multi-segment linear constitutive model described in Section 2.2 incorporates stochastic functions through a Weibull distribution to define parameter variability. The density function of the Weibull distribution is shown in Equation (20).

(20) $$f\left(x;k,\lambda ,loc\right)=\frac{k}{\lambda }{\left(\frac{x-loc}{\lambda }\right)}^{k-1}\exp\left[-{\left(\frac{x-loc}{\lambda }\right)}^{k}\right]$$

where x is a variable that satisfies the Weibull distribution (amplification factor of the elastic segment energy of the bond), k is the shape parameter, λ is the scale parameter, and loc is the position parameter. The expected value μ and standard deviation $s_{d}$ of the energy amplification coefficient x are determined by Equation (21).

(21) $$\mu =loc+\lambda \cdot \Gamma \left(1+\frac{1}{k}\right);s_{d}=\lambda {\left[\Gamma \left(1+\frac{2}{k}\right)-\Gamma^{2}\left(1+\frac{1}{k}\right)\right]}^{1/2}$$

where $\Gamma \left(x\right)$ represents the gamma function simulated using a well-known numerical procedure.

**Figure 10:**
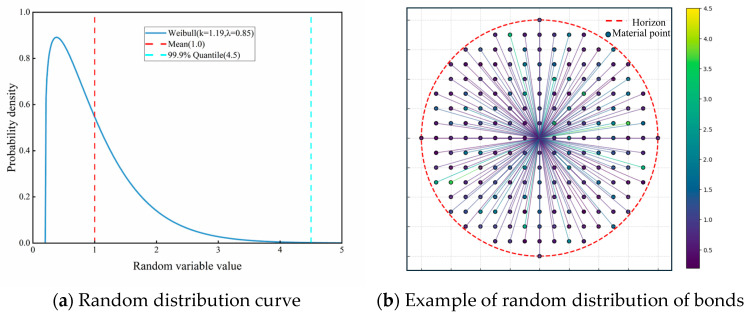
Probability density distribution of random coefficients and examples of random arrangements.

To standardize peridynamic parameter calibration, the random energy scaling coefficients for the elastic segments of both the mortar and the interfacial transition zone (ITZ) are modeled using a Weibull distribution with a bias of 0.2, a 99.9% lower quantile of 4.5, and an expected value of 1.0. Figure 10 illustrates the resulting probability density functions and stochastic bond parameter distributions.

## 4. Verification and Discussion of Results

### 4.1. Setting Model and Material Parameters

**Figure 11:**
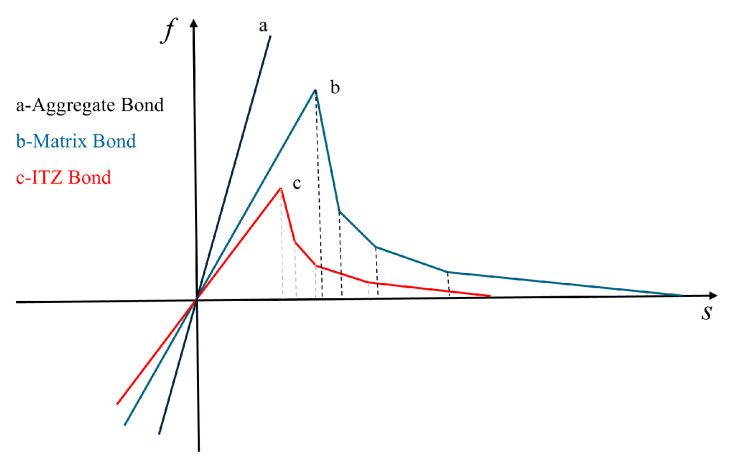
Bond force functions of different types of bonds.

Building on the proposed methodology, we developed a mesoscale concrete model incorporating fully graded aggregate distributions and multi-phase material constituents. The model includes four distinct bond types: intra-mortar, intra-aggregate, mortar-aggregate interface, and inter-aggregate bonds. As illustrated in Figure 11, mortar and ITZ bonds follow the multi-linear constitutive law described in Section 2.2, while inter-aggregate bonds are assigned the same mechanical response as the ITZ bonds.

This study employs five multi-linear segments to characterize the complete stress-strain response of full-grade aggregate concrete under uniaxial tension [38]. Among the five linear intervals, the first represents the elastic region. The subsequent four segments have stiffness degradation ratios of 1/20,1/40,1/225,1/850, and corresponding force density reduction factors of 0.5,0.15,0.15,0.2, respectively. These parameters yield a calculated γ value of 72.66. The stiffness degradation ratios and load decay coefficients were initially derived from experimental data characterizing the complete stress–strain response of fully graded concrete and were subsequently calibrated through iterative peridynamic (PD) simulations to ensure the convergence of the constitutive parameters.

**Figure 12:**
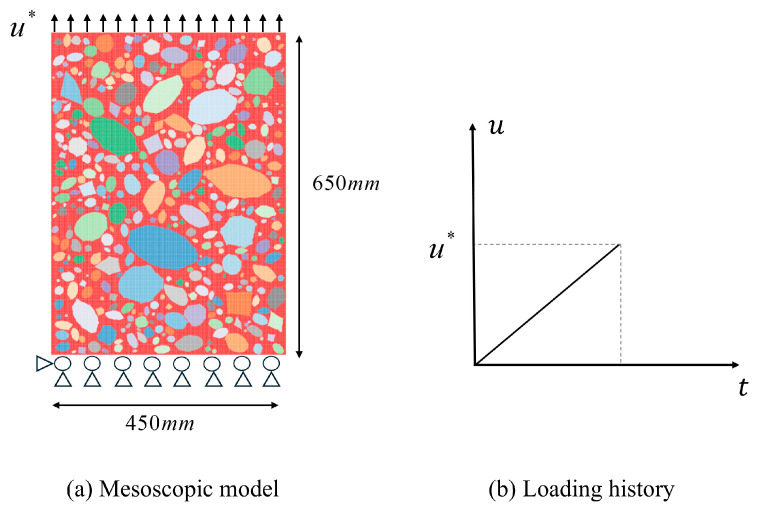
Mesoscopic model and boundary conditions.

The comparative experiment adopted a uniaxial tensile loading method. To ensure an uniform transmission of the load into the specimen, bolts were embedded at both ends as loading points. No bolts were installed within the 650 mm central measurement section of the specimen. Accordingly, to facilitate comparison with the experimental configuration, a simplified two-dimensional model was adopted. The established model had a cross-sectional dimension of 450 mm × 650 mm, with an aggregate volume fraction of 70%. To avoid the generation of excessively elongated (needle-shaped) or flat (flake-shaped) particles, the axial ratio of the aggregates is restricted to the range (1.0, 2.0), and the angularity coefficient to the range (1.05, 1.45). Both parameters are randomly assigned during aggregate generation [39]. Table 2 lists the parameter settings used in the peridynamics simulations, while Table 3 summarizes the basic mechanical properties of each model component. Where applicable, the bond parameters for mortar bonds, interface bonds, and aggregate phases are adopted from [40,41]. Notably, the energy release rate used to define the softening stage was selected from the parameter range summarized by Wu [40] and further calibrated using experimental mechanical response curves [38]. The numerical experiment was performed under displacement-controlled loading, with the specific boundary conditions illustrated in Figure 12. The axial movement of the specimen’s lower boundary was constrained, the left endpoint was fixed, and a uniform displacement with a specified increment was applied to the upper boundary along the positive Y-axis.

### 4.2. Impact of Loading Rate

**Figure 13:**
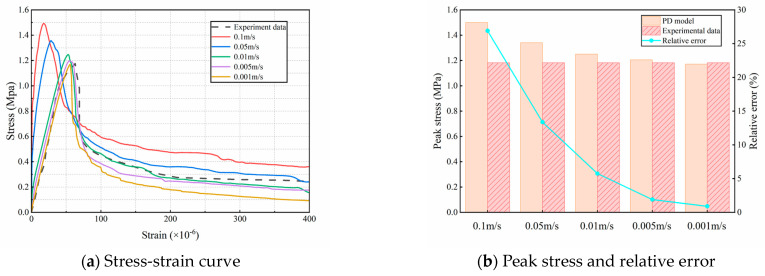
Stress-strain curves and tensile strength of simulated experiments under different loading rates.

**Figure 14:**
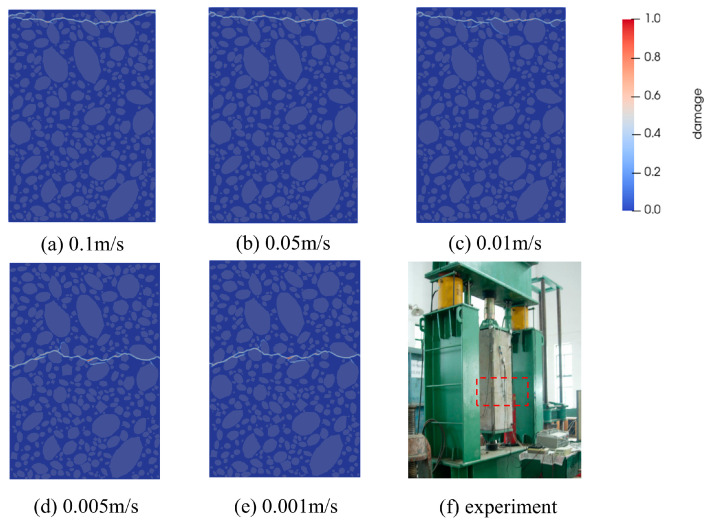
Final fracture of the model under different loading speeds.

Previous studies have demonstrated that the loading rate significantly affects crack propagation patterns and the stress–strain behavior of concrete [42]. To identify an optimal loading rate that balances numerical accuracy and computational efficiency, this study investigates the effect of loading rate on crack propagation and stress–strain behavior under various dynamic loading conditions. Loading rates of 0.1 m/s,  0.05 m/s,  0.01 m/s, 0.005 m/s, and 0.001 m/s were selected for dynamic loading simulation. Simulation results were then compared and validated against the experimental data [38], using both the stress–strain curve and final fracture morphologies of the specimens, as shown in Figure 13 and Figure 14.

Figure 13a presents the stress–strain relationships under different loading rates, showing that the slope of the elastic segment stabilizes as the loading rate decreases, while the peak load gradually diminishes. At loading rates v>0.005 m/s, the dynamic strength effect of concrete shows high peak stress and low ductility due to the fast loading rate; when v≤0.005 m/s, the slope of the stress-strain curve as well as the peak load remain basically unchanged, which also maintains a high degree of overlap with the experimental stress-strain curve. This suggests that the loading rate of v≤0.005 m/s satisfies quasi-static loading conditions, resulting in more stable mechanical responses under uniaxial tension. As shown in Figure 13b, at a loading rate of 0.005 m/s, the simulated peak stress is 1.205 MPa, with a relative error of 1.9% compared to the experimental result. At a loading rate of 0.001 m/s, the simulated peak stress is 1.171 MPa, with a relative error of 0.9%, also within the acceptable range. As shown in Figure 14, at high loading rates, the material exhibits brittle fracture behavior. The critical bond fracture threshold is reached rapidly, and crack tip dynamic effects—such as inertia—become significant. Crack bifurcation is suppressed, and the rapid energy input promotes the propagation of a single dominant crack. Consequently, the system generally follows a path that minimizes energy dissipation, resulting in path stabilization. At low loading rates, damage evolution is more pronounced, characterized by a larger microdamage zone near the crack tip and an increased likelihood of bifurcation. Local stress fluctuations and microdefect activation can initiate multiple competing crack paths, causing bifurcations or irregular extensions (path destabilization) as energy is gradually released. The fracture locations in specimens subjected to low loading rates more closely matched the experimental observations. Overall, the tensile strength of fully graded concrete is significantly influenced by the loading rate, and our study’s findings align well with the observed experimental behavior [43]. The last two loading rates show smaller deviations from the experimental results, and the crack morphology closely matches the fracture patterns observed in laboratory tests. However, applying a loading rate of 0.001 m/s significantly increases computational time. To ensure simulation stability and maximize computational efficiency, a uniform loading rate of 0.005 m/s was adopted in subsequent uniaxial tensile strength simulations of fully graded concrete.

### 4.3. Effect of Aggregate Randomness on Uniaxial Tensile Properties

**Figure 15:**
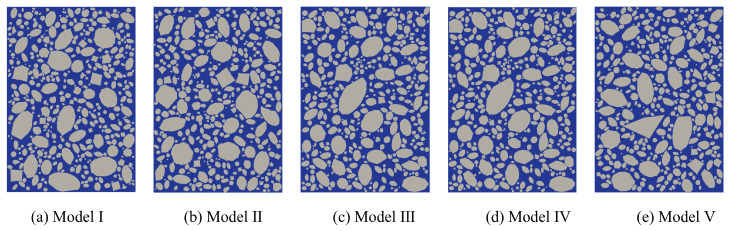
Fine view of the concrete model with random aggregate distribution.

To account for the impact of random aggregates on the numerical results, five fine-scale models with a 70% aggregate volume ratio were established to simulate concrete specimens under uniaxial tension, as shown in Figure 15.

**Figure 16:**
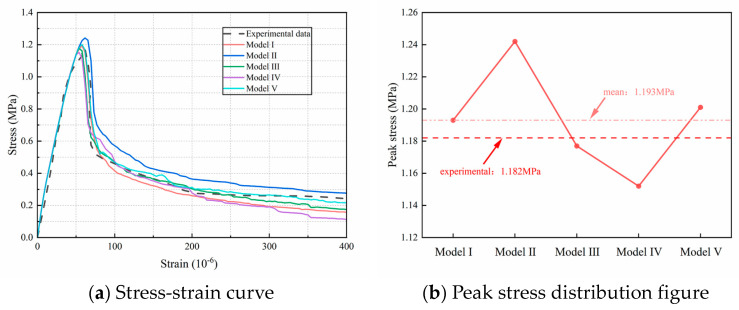
Tensile stress-strain full curve and peak stress distribution of fully graded concrete with different random aggregates.

Figure 16 presents the complete stress-strain curves for the fine-scale models with varying random aggregate distributions, along with the corresponding peak stress values. Figure 16a shows the overall stress-strain curve obtained from uniaxial experiments on concrete specimens [38]. The curve reveals that each fine-scale model undergoes elastic, elasto-plastic, plastic, and softening phases during uniaxial loading. This suggests that the overall mechanical response of each concrete specimen was accurately captured using a multi-segment linear principal structure, aligning well with the available experimental curves. Figure 16b illustrates that variations in random aggregate distributions affect the peak stress outcomes of the simulations. This effect is primarily attributed to the stochastic variability of aggregate distributions, which alters spatial configurations—such as particle position, geometry, and size—and consequently affects the strain energy distribution within the concrete matrix. Table 4 presents the peak stress values corresponding to each model, with a maximum of 1.242 MPa and a minimum of 1.152 MPa, resulting in a range of 0.09 MPa and a relative range of 7.81%, indicating a certain degree of variation among the peak values. However, the overall coefficient of variation (CV) is 2.78%, indicating low variability in peak stress. The relative error between the average peak stress of the five models and the experimental value is 0.93%, which falls within an acceptable range, indicating that using five model samples in the simulation effectively reduces the impact of random aggregate distribution on the simulation results.

**Figure 17:**
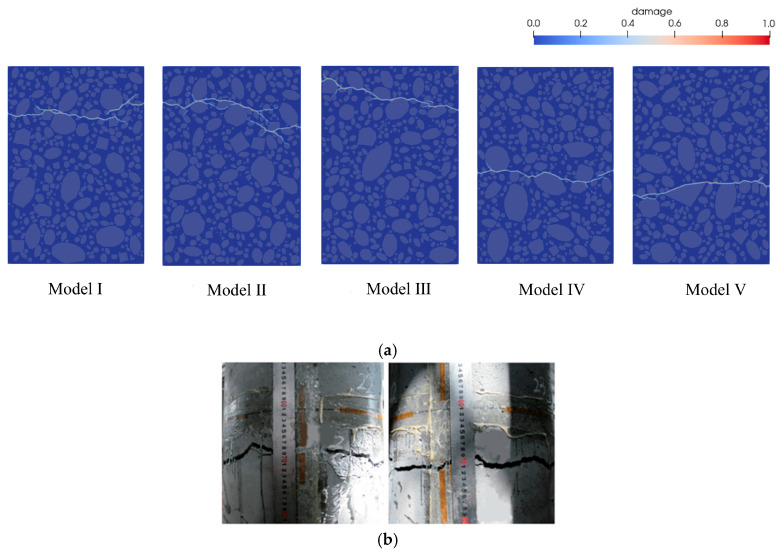
Fracture diagrams of specimens; (**a**) final crack extension pattern for fine-scale models with different randomly distributed aggregates, (**b**) experimental [43].

Figure 17a presents the final crack propagation patterns of models with varying random aggregate distributions, while Figure 17b displays the corresponding experimental crack patterns [43]. As shown in Figure 17a, the simulated crack propagation under tensile loading closely matches the experimental observations, demonstrating the predictive capability of the peridynamics framework. Fracture locations are also observed to vary depending on the random aggregate distribution. However, the overall crack propagation patterns exhibit consistent characteristics, with cracks predominantly initiating and propagating along the boundaries of large aggregates, consistent with experimental findings [44].

**Figure 18:**
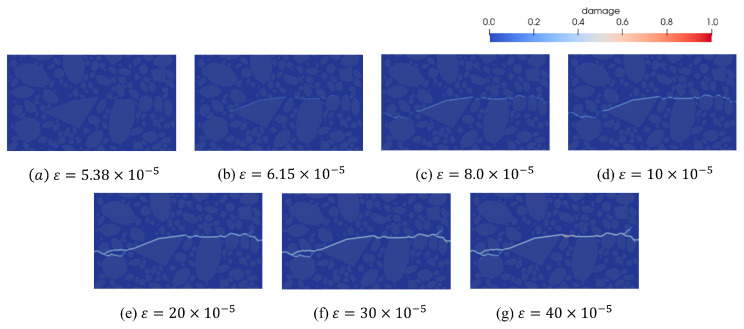
Concrete model V expansion process under uniaxial stretching.

To better visualize the internal crack evolution, Figure 18 illustrates the crack expansion forms corresponding to each strain stage of the concrete Model V under uniaxial tension. The specimen reaches its ultimate tensile strength at $\varepsilon =6.15\times 10^{-5}$, as shown in Figure 18b. Initial damage appears in the ITZ and gradually progresses with increasing external load. At a strain of ε = 5.38 × 10^−5^, just before reaching peak stress, microcracks began to form within the large aggregate regions. Furthermore, the ascending portions of the mechanical response curves are nearly identical across models at this stage, indicating that the aggregate distribution has minimal influence on the elastic-phase mechanical behavior. After peak stress, microcracks propagate and coalesce along the aggregate interfaces, eventually forming major cracks that penetrate the specimens. During crack propagation, the presence of oversized aggregates significantly influences the direction of crack initiation and growth, as the aggregate strength is greater than that of the surrounding mortar matrix and ITZ. As a result, cracks initially form at the aggregate interfaces and propagate along the aggregate boundaries. Therefore, the presence and spatial distribution of oversized aggregates influence crack development, contributing to the variability observed in the softening phase of the stress–strain curve.

### 4.4. Effect of Aggregate Axial Ratio on Tensile Properties

The axial ratio of aggregates significantly influences the internal stress distribution in concrete, thereby influencing microcrack persistence, damage accumulation, and progression to macroscopic failure. Experimental studies by Deng [39], Ichino [45], and others demonstrated that the inclusion of elongated or lamellar aggregates has a more pronounced effect on concrete strength. Hong Li et al. [34] employed numerical simulation techniques to investigate the weakening effect of axial ratio on the tensile strength of concrete. These findings suggest that the axial ratio of aggregates is an essential factor influencing the tensile strength of fully graded concrete and should not be overlooked in related studies.

**Figure 19:**
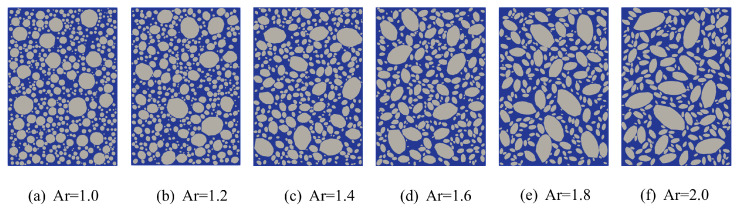
Fully graded concrete specimens with different Ar.

To investigate the influence of aggregate axial ratio on the tensile behavior of fully graded concrete, six sets of uniaxial tensile specimens with varying axial ratios were developed. The axial ratios of the six fully graded concrete models ranged from 1.0 to 2.0 in increments of 0.2, with identical aggregate volume fractions across all specimens. To eliminate the influence of the angularity coefficient on tensile performance and better simulate realistic aggregate geometries, the angularity coefficient was uniformly set to 1.05. All other parameters were kept consistent with the previous study, and the corresponding model configuration is shown in Figure 19.

**Figure 20:**
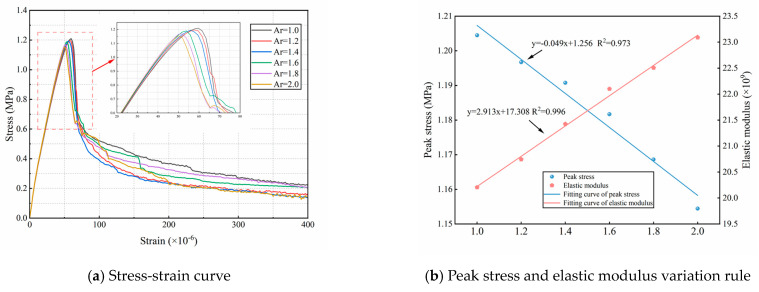
Tensile stress-strain curves with corresponding peak stress and elastic modulus for different Ar.

Figure 20a presents the stress–strain curves for various aggregate axial ratios. As the axial ratio increases, the slope of the stress–strain curve becomes steeper, while the peak stress exhibits a decreasing trend. Table 5 summarizes the peak stress values corresponding to each aggregate axial ratio. As shown in Figure 20b, the ultimate tensile strength of concrete specimens exhibits a clear linear relationship with the aggregate axial ratio. From Ar = 1.0 to Ar = 2.0, the peak stress decreased by 4.15%, while the elastic modulus increased by 14.31%. The reduction in ultimate tensile strength is primarily due to stress concentration at the tips of slender aggregates, which promotes early microcracking. In addition, aggregates with higher axial ratios possess larger surface areas, increasing the proportion of the ITZ and the likelihood of internal defects, thereby reducing the overall specimen strength. The increase in elastic modulus suggests that a higher axial ratio effectively enhances concrete stiffness. This is because aggregates typically possess a higher modulus of elasticity than the cement matrix. Moreover, a greater projected length along the tensile direction allows aggregates to more effectively utilize their stiffness, thereby constraining matrix deformation and enhancing the overall tensile modulus of the composite.

**Figure 21:**
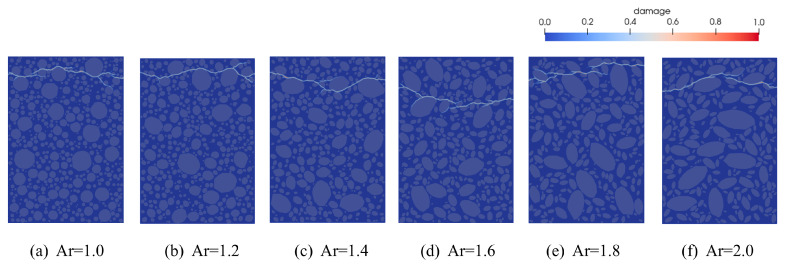
Final crack propagation in concrete with different Ar content.

Figure 21 illustrates the simulated fracture behavior of concrete under uniaxial tension using peridynamic theory, considering various aggregate axial ratios (Ar). The corresponding damage cloud patterns for Ar values ranging from 1.0 to 2.0 are compared. The results indicate that variations in the aggregate axial ratio significantly influence the internal stress distribution and crack propagation patterns. As the Ar value increases, the periphery of elongated aggregates gradually becomes the primary region of crack extension. Fractures begin to appear more frequently along aggregate boundaries, and the crack paths exhibit increased complexity. At low axial ratios (Ar = 1.0–1.4), the aggregate distribution is denser, and cracks preferentially propagate along aggregate interfaces. It is easier for cracks to develop within the concrete and form a coherent main crack. At high axial ratios (Ar = 1.6–2.0), increased aggregate spacing causes cracks to propagate more through the mortar matrix, forming crack paths that traverse aggregates. Meanwhile, crack propagation within the mortar becomes more difficult, resulting in discontinuous and fragmented crack patterns.

### 4.5. Effect of Aggregate Angularity Coefficient on Tensile Properties

**Figure 22:**
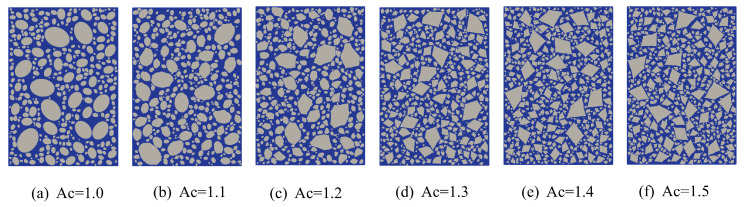
Fully graded concrete specimens with different Ac.

Aggregates used in hydraulic concrete with large aggregate content are predominantly crushed stone and are commonly simplified as polygons in two-dimensional simulations. However, this simplification overlooks the angularity of the aggregates and fails to account for its influence on the mechanical behavior of concrete. Gu et al. [33] experimentally demonstrated that aggregate angularity influences the splitting tensile strength of concrete; however, the range of angularity coefficients considered in their study was limited. To investigate the influence of aggregate angularity on the tensile strength and fracture behavior of fully graded concrete, numerical specimen models were constructed with angularity coefficients of 1.0, 1.1, 1.2, 1.3, 1.4, and 1.5, as shown in Figure 22. As shown in the model, increasing angularity results in sharper aggregate geometries and a notable increase in aggregate surface complexity and packing density. To eliminate the influence of axial ratio on the simulation results, a fixed value of 1.4 was assigned to the aggregate axial ratio, based on the average value measured by Huang et al. [46] for fully graded aggregates. All other phase parameters remained consistent with those used in the previous analysis.

**Figure 23:**
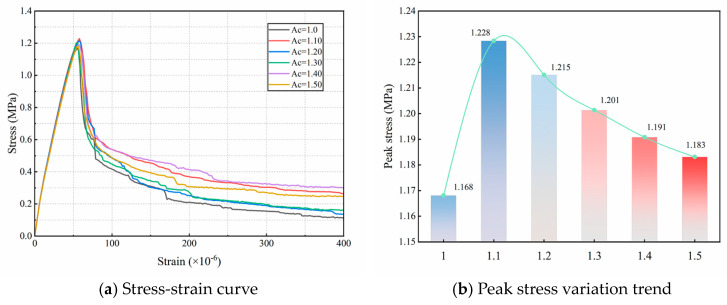
Corresponding tensile stress-strain relationship and peak stress trend with different Ac.

Figure 23a presents the simulated stress–strain curves, which exhibit similar overall profiles but differ in the softening phase and peak tensile strength. Figure 23b illustrates the variation in peak tensile strength with respect to different angularity coefficients. The peak tensile strength of the concrete initially increases with angularity, reaches a maximum, and then gradually decreases. This trend demonstrates that the peak tensile strength increases with angularity up to a certain point, then decreases. When Ac = 1.10, the tensile strength is at its maximum of 1.228 MPa, an increase of 5.14%, after which it gradually decreases. At Ac = 1.50, the tensile strength decreases to its minimum, corresponding to only a 1.28% increase relative to that at Ac = 1.0. The main reason is that at Ac = 1.0, the aggregate model is elliptical, and the smooth contact surfaces promote crack initiation, leading to the lowest tensile strength. As the angularity increases, the surface roughness and contact area between the aggregates and mortar both increase, enhancing the mechanical interlocking effect. This significantly improves the peak tensile strength of fully graded concrete. The main reason for the decrease in peak stress is that higher Ac results in more irregular aggregate shapes with an increased number of sharp corners, which promote stress concentration and thereby reduce the tensile strength of the concrete. Under a constant volume fraction, the density of concrete aggregates increases, leading to a larger total ITZ and weaker interfaces, which reduce the overall strength. However, the increased number of aggregates also hinders crack propagation, resulting in a tensile strength that remains higher than that at the lowest Ac. Overall, increasing the angularity coefficient can moderately enhance the tensile strength of fully graded concrete.

**Figure 24:**
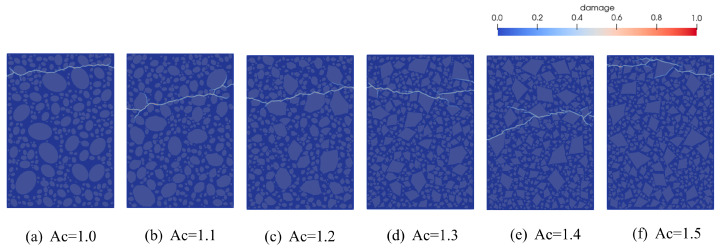
Final crack propagation in concrete with different Ac content.

To better illustrate the evolution of damage and fracture patterns, Figure 24 presents the final damage cloud distributions for specimens with different levels of aggregate angularity. It is evident from the figure that aggregate angularity exerts a significant influence on crack propagation behavior in concrete. When Ac < 1.30, the stress distribution is relatively uniform, and cracks tend to propagate along straighter paths with fewer branches, often bypassing aggregates within the matrix. When Ac ≥ 1.30, cracks exhibit frequent bifurcations and zigzag paths, as they are forced to deviate or split upon encountering sharp aggregate edges. This phenomenon is attributed to the gradual increase in the angularity coefficient, which transforms aggregate shapes from smooth to sharp, intensifying stress concentrations within the concrete. As a result, microcracks form and propagate more readily. Simultaneously, the internal aggregate density increases, contributing to a higher number of microcracks under external loading. However, the aggregates can also impede crack propagation, leading to dispersed crack paths and promoting crack bridging.

## 5. Conclusions

This study develops a peridynamic constitutive model that incorporates tensile softening behavior to investigate the complete stress–strain response of concrete under uniaxial tension. Additionally, two intrinsic aggregate characteristics—axial ratio and angularity—are considered in the development of an efficient microstructure generation method that accurately captures their geometric features. This method was subsequently applied to construct a microfracture model for fully graded concrete. The enhanced peridynamic (PD) model is validated through simulations incorporating the generated microstructure, and the effects of aggregate angularity and axial ratio on the tensile behavior of fully graded concrete are systematically analyzed and discussed. The main conclusions are as follows:

1. The proposed peridynamic model accurately simulates the complete stress–strain response of fully graded concrete under uniaxial tension, encompassing the elastic phase, crack initiation, propagation, and final failure. Moreover, phenomena such as crack initiation, coalescence, bridging, and branching are naturally captured without requiring predefined failure criteria or remeshing, offering a robust and reliable approach for simulating the entire tensile failure process of concrete.
2. The peridynamic simulation results reveal pronounced strain rate-dependent behavior. As the loading rate decreases, concrete ductility decreases while tensile strength approaches a stable value. At higher loading rates, fracture tends to initiate near the loading point, whereas at lower rates, cracks propagate preferentially along pre-existing weak zones. A loading rate of v≤ 0.005 m/s is sufficient to satisfy quasi-static loading in simulation.
3. The numerical model of fully graded concrete based on randomly generated aggregate morphology exhibits noticeable variability in its results. This variability stems from the irregular shapes and spatial distributions of aggregates, which strongly affect internal force transmission mechanisms. Therefore, to minimize the impact of aggregate randomness, at least five realizations should be employed in both numerical simulations and physical experiments.
4. As the axial length-to-radius ratio (Ar) of aggregates increases, the slope of the elastic segment of the tensile stress–strain curve increases, whereas the peak stress decreases. An increased Ar modifies the spatial distribution of the ITZ, leading to a concentration of interface bonds, which adversely affects the fracture behavior of the specimen. Therefore, moderately reducing the aggregate Ar may facilitate a more favorable and uniform stress distribution in fully graded concrete.
5. As the angularity coefficient (Ac) of the aggregates increased, the ultimate tensile strength of fully graded concrete specimens initially increased, peaking at Ac = 1.10, before gradually decreasing. Crack propagation progressively transitioned from a single isolated crack to a dominant crack accompanied by multiple branches, with the crack path becoming increasingly tortuous. Overall, increasing Ac can enhance the tensile strength of fully graded concrete to a certain extent; however, excessive angularity does not necessarily result in further improvement. Therefore, selecting an appropriate angularity coefficient is essential for optimizing the tensile performance of fully graded concrete.

Although this study has achieved certain results, some limitations remain and warrant further investigation in future work. The improved PD model and aggregate generation method proposed in this study are primarily based on a two-dimensional plane and have not yet fully considered the morphological characteristics of aggregates in three-dimensional space. In the future, a method for efficiently generating high-fidelity aggregate models in three-dimensional space will be developed, in order to further investigate their impact on the mechanical properties of concrete.

## Figures and Tables

**Table 1:** Relationship between Ac and indentation depth magnification coefficient.

Ac Range Interval	Minimum Concavity Magnification Factor	Maximum Concavity Magnification Factor
Ac<1.26	0.0	$0.2533+3.458/S_{c}$
1.26≤Ac<1.35	0.0	$2.40-0.08\left(S_{c}-1\right)$
1.35≤Ac<1.40	0.0	$2.45-0.35\left(S_{c}-1\right)$
Ac≥1.40	0.0	$2.30-0.4\left(S_{c}-1\right)$

**Table 2:** Calculation parameters for the peridynamics model.

Parameters	Label	Value
Size of material point $\left(mm\right)$	∆x	1
Horizon $\left(mm\right)$	δ	3.15∆x
Time step $\;\left(s\right)$	∆t	$1\times 10^{-7}$
Final displacement $\;\left(m\right)$	$u^{*}$	$3\times 10^{-4}$

**Table 3:** Mechanical parameters of components in the full-graded concrete fine-scale model.

Class	Density ρ/kg·m	Young’s Modulus E/GPa	Poisson Ratio ν	$G_{1}/N\cdot m^{-1}$	$G_{f}/N\cdot m^{-1}$
Aggregate	2700	50	0.33	-	-
Mortar	2000	15	0.33	0.70	50.9
ITZ	-	10	0.33	0.22	16

**Table 4:** Comparison of numerical simulation and experimental tensile peak stresses for different aggregate concrete models.

Class	Experiment	Model I	Model II	Model III	Model IV	Model V	Mean
Peak stress (MPa)	1.182	1.195	1.242	1.177	1.152	1.201	1.193
Relative error	-	1.10%	5.08%	−0.42%	−2.54%	1.61%	0.93%

**Table 5:** The angularity coefficient corresponds to peak stress and elastic modulus.

Ar	1.0	1.20	1.40	1.60	1.80	2.0
Peak stress (MPa)	1.204	1.198	1.191	1.182	1.169	1.154
Elastic modulus (×10^9^)	20.208	20.743	21.424	22.099	22.507	23.089

## Data Availability

The raw data supporting the conclusions of this article will be made available by the corresponding author on request.
